# Cytokines and tryptophan metabolites can predict depressive symptoms in pregnancy

**DOI:** 10.1038/s41398-022-01801-8

**Published:** 2022-01-26

**Authors:** Qiong Sha, Zach Madaj, Sarah Keaton, Martha L Escobar Galvis, LeAnn Smart, Stanislaw Krzyzanowski, Asgerally T. Fazleabas, Richard Leach, Teodor T. Postolache, Eric D. Achtyes, Lena Brundin

**Affiliations:** 1 Department of Neurodegenerative Science, Van Andel Institute, Grand Rapids, MI USA; 2 Bioinformatics & Biostatistics Core, Van Andel Institute, Grand Rapids, MI USA; 3 Division of Psychiatry & Behavioral Medicine, Michigan State University College of Human Medicine, Grand Rapids, MI USA; 4 Pine Rest Christian Mental Health Services, Grand Rapids, MI USA; 5 Department of Obstetrics, Gynecology and Reproductive Biology, College of Human Medicine, Michigan State University, MI USA; 6 Mood and Anxiety Program, Department of Psychiatry, University of Maryland School of Medicine, Baltimore, MD USA; 7 Rocky Mountain MIRECC for Suicide Prevention, Aurora, CO USA; 8 Military and Veteran Microbiome Consortium for Research and Education, Aurora, CO USA; 9 Capital MIRECC, VISN 5, Baltimore, MD USA

**Keywords:** Predictive markers, Depression

## Abstract

Depression during and after pregnancy affects up to 20% of pregnant women, but the biological underpinnings remain incompletely understood. As pregnancy progresses, the immune system changes to facilitate fetal development, leading to distinct fluctuations in the production of pro-inflammatory factors and neuroactive tryptophan metabolites throughout the peripartum period. Therefore, it is possible that depression in pregnancy could constitute a specific type of inflammation-induced depression. Both inflammatory factors and kynurenine metabolites impact neuroinflammation and glutamatergic neurotransmission and can therefore affect mood and behavior. To determine whether cytokines and kynurenine metabolites can predict the development of depression in pregnancy, we analyzed blood samples and clinical symptoms in 114 women during each trimester and the postpartum. We analyzed plasma IL-1*β*, IL-2, -6, -8, -10, TNF, kynurenine, tryptophan, serotonin, kynurenic- quinolinic- and picolinic acids and used mixed-effects models to assess the association between biomarkers and depression severity. IL-1*β* and IL-6 levels associated positively with severity of depressive symptoms across pregnancy and the postpartum, and that the odds of experiencing significant depressive symptoms increased by >30% per median absolute deviation for both IL-1*β* and IL-6 (both *P* = 0.01). A combination of cytokines and kynurenine metabolites in the 2nd trimester had a >99% probability of accurately predicting 3rd trimester depression, with an ROC AUC > 0.8. Altogether, our work shows that cytokines and tryptophan metabolites can predict depression during pregnancy and could be useful as clinical markers of risk. Moreover, inflammation and kynurenine pathway enzymes should be considered possible therapeutic targets in peripartum depression.

## Introduction

Peripartum depression (PPD) is defined as depression with onset during and up to four weeks after delivery [1]. In addition to symptoms of depression, which can range from mild to severely incapacitating, anxiety and suicidality are also frequently reported [2]. PPD in the mother is linked to low birth weight and prematurity [3], as well as emotional problems in the offspring, spanning from early childhood to adolescence [4]. Selective serotonin reuptake inhibitors (SSRIs) are the most common treatment for pregnancy-related depression today, but they have been shown only to be effective in approximately 50% of patients [5]. In addition, SSRIs are associated with certain risks during pregnancy, such as discontinuation syndrome in the newborn [6] and postpartum hemorrhage in the mother [7]. Physicians and mothers may therefore be reluctant to the use SSRIs, leaving this condition un- or undertreated.

The biological mechanisms underlying depressive symptoms during pregnancy remain to be fully elucidated. Several contributing factors have been suggested, including dysregulation of hormones, immune response, and aberrant genetic, or epigenetic factors [8]. Additionally, an increasing body of evidence suggests a role for inflammation in depression in general, and possibly specifically in PPD [9]. Due to the profound changes that the immune system undergoes during pregnancy it has been suggested that inflammation could play a particularly important role in the development of depressive symptoms during this period [2, 10]. Several recent studies have shown an association between levels of pro-inflammatory cytokines in the blood of pregnant women and depressive symptoms [11–13].

**Fig. 1:**
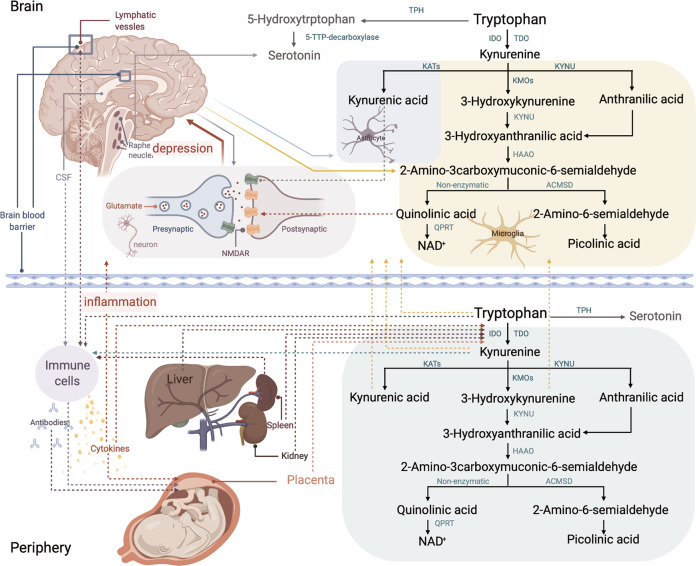
Tryptophan metabolic pathways in the brain and periphery. Abbreviations: TPH tryptophan hydroxylase, IDO indoleamine 2,3-dioxygenase. TDO tryptophan 2,3-dioxygenase. KATs kynurenine aminotransferases. KYNU kynureninase, HAAO 3-hydroxyanthranilate 3,4-dioxygenase, ACMSD aminocarboxymuconate-semialdehyde decarboxylase, QPRT quinolinic acid phosphoribosyltransferase, CSF cerebrospinal fluid, NMDAR *N*-methyl-D-aspartate receptor, NAD nicotinamide.

The kynurenine pathway is the major enzymatic pathway that catabolizes tryptophan and has considerable immunomodulatory activity (Fig. 1). Interestingly, kynurenine pathway enzymes are highly expressed in the placenta, where their activity is key in the regulation of the maternal-fetal immune response [14]. The expression of indolamine-2,3-dioxygenase (IDO), the enzyme that catalyzes the transformation of tryptophan to kynurenine, as well as other pathway enzymes, increases with placental development [15]. In addition, it is known that the activity of the pathway is increased by interferon-*α* and IL-6 [16]. Changes in activity are important because several kynurenine pathway metabolites are neuroactive, including quinolinic acid (QUIN) and kynurenic acid (KYNA), both impacting glutamate neurotransmission [17]. Increased pathway activity could also affect serotonin levels, as tryptophan is also the substrate for its production (Fig. 1). Thus, it is plausible that changes in kynurenine pathway activity (and its metabolite levels) are causally linked to the generation of depressive symptoms as they can influence both central glutamatergic and serotonergic- mechanisms [18].

In spite of data pointing to an involvement of inflammatory mechanisms in pregnancy-related depression, longitudinal studies assessing how levels of inflammatory factors and kynurenine pathway metabolites fluctuate during pregnancy are sparse. Also, it is not known whether an increased production of these molecules can predict the development of depressive symptoms. Such information would be important for developing biomarkers to help clinicians identify women at risk, possibly even before symptoms manifest. We hypothesized that the levels of pro-inflammatory cytokines and neuroactive kynurenine metabolites would be positively linked to the severity of depressive symptoms, and that single markers, or a combination thereof, measured early in pregnancy could serve as biomarkers to predict subsequent depression.

## Materials and Methods

### Study design

This study was approved by the Institutional Review Board (IRB) of Michigan State University, East Lansing, with reciprocal approvals by Van Andel Research Institute IRB and Spectrum Health IRB, both in Grand Rapids, Michigan. Informed consent was obtained from all subjects. Pregnant women were enrolled in their first trimester, returning each trimester and in the postpartum for psychiatric assessments and blood sampling. Inclusion criteria for enrollment were: all races and national origins of pregnant females, age 18 and above, English speaking and able to give informed consent as well as able to comply with and complete study procedures. Our exclusion criteria were: nonpregnant females, patients with psychotic symptoms and/or severe cognitive impairment that interfere with their ability to give informed consent or to complete study assessments. Patients that could not read and write in English as research measures used have only been validated in English-speaking populations. Patients that had blood-borne chronic infections including hepatitis B, C, or HIV as established at routine pregnancy blood screens. Patients who had any schizophrenia spectrum disorder or bipolar disorder type 1 (based on self-report and SCID interview). Patients who reported ongoing substance abuse or dependence (in the past 3 months). Three patients had a systemic autoimmune disease (systemic lupus erythematosus and myasthenia gravis) and were subsequently excluded, leaving 114 women in the final cohort.

### Clinical cohort

The women were recruited from the Obstetrics and Gynecology clinic at Spectrum Health, Grand Rapids, Michigan, between 2015-2018. The mean age of the women was 25 (±5.9) at the beginning of the study. All the participants had high school level education or above, with 16% obtaining a bachelor’s degree. More than 60% of the women lived in a household with an annual income of less than $34,000. Our cohort was ethnically diverse, with over 50% non-White/Caucasian. Caucasian was the largest individual group at 47%, followed by Black/African American at 26%. We also had study participants of American Indian, Alaskan Indian and multiracial ethnicity (Table 1). The majority of the women in the study were single or unmarried; 40% were married or living with someone (Table 1); 42% of the women had no previous children in the household, 30% had one child and the rest of the participants had two to five children.

**Table 1:** Patient demographics.

	Median/ Frequency	SD %
**Age**	25	±5.9
**BMI**	29	±8.9
**Taking an SSRI (%)**	4	3.5%
**Employed %**	64	56.1%
**Race**		
American Indian or Alaskan Indian	2	1.8%
White/Caucasian	54	47.4%
Black or African American	30	26.3%
Multiracial	12	10.5%
Other	16	14%
**Highest education**		
Some high school	19	16.7%
High school diploma	31	27.2%
Technical/ trade/ allied health training degree or certification	5	4.4%
Some college	41	36%
Bachelor’s degree or higher	18	15.8%
**Annual household income**		
$15,000 or less	41	36%
$15,001–$34,000	31	27.2%
$34,001–$70,000	21	18.4%
$70,001–$120,000	10	8.8%
$120,000 or more	8	7.0%
**Marital status**		
Married or living with someone	46	40.4%
Single, unmarried	59	51.8%
Have a partner, not living together	8	7.0%

### Patient evaluation

The Edinburgh Postnatal Depression Rating Scale (EPDS) was used to measure depressive symptom severity [19] at each of the four visits. The Structured Clinical Interview for DSM-IV (SCID) was administered by a trained clinician at baseline, and then again whenever a subject scored >10 on the EPDS, to establish (or rule out) a new diagnosis of major depressive disorder with peripartum onset (PPD), as defined by DSM-5 [20]. An EPDS score ≥ 13 over the last 7 days has been shown to be a valid indicator of depression during pregnancy and the first postpartum year [21] and was thus used as dichotomous variable (EPDS ≥ 13 and < 13) in this study. A somatic health report, including comorbid medical conditions, medications, vital signs (BMI, pulse, blood pressure, temperature), and a blood draw were also completed at all visits.

### Plasma cytokine analysis

Blood samples were collected by venous puncture into EDTA tubes, between 9:00 am and 12:00 pm and immediately transported to the laboratory on ice. Samples were centrifuged into plasma (700 × *g* at 4 °C), aliquoted and immediately frozen at –80 °C until analysis. IL-1*β*, IL-2, IL-6, IL-8, IL-10, and TNF levels were run on a Sector 600 (K15049D, Meso Scale Diagnostics, Rockville, MD, USA) following manufacturer’s instructions as previously published [2]. Samples were run in duplicate and mean values were used for analysis. Inter-assay coefficients of variation (% CV) were: IL-1*β* (3.5%), IL-2 (3.0%), IL-6 (2.2%), IL-8 (2.4%), IL-10 (1.8%), and TNF (2.8%).

### Detection of tryptophan, serotonin, and kynurenine pathway metabolites

The laboratory analyses were performed by staff that were blinded for all clinical parameters. A research number was assigned to each sample to enable future integration with clinical data. Tryptophan (TRP), serotonin (SERO), kynurenine (KYN), KYNA, QUIN and picolinic acid (PIC) were analyzed as previously described [2]. Briefly, plasma TRP, SERO and KYN concentrations were measured using high-performance-liquid chromatography with ultraviolet/visible wavelength detection and results were analyzed by Chromeleon 7.2 Chromatography Data System (Thermo Scientific, Dionex). QUIN and PIC were separated by gas chromatography-mass spectrometry (GC-MS) and quantified using Thermo Xcaliber and Waters Masslynx software. Ultra-high-performance-liquid-chromatography, coupled to mass spectrometry (UPLC-MS/MS) was used to measure KYNA concentrations. Analytical conditions were set according to a previously published protocol [22]. Three derivative predictors—metabolite ratios—were created by division, KYN/TRP (rKT), QUIN/KYNA (rQK) and QUIN/PIC (rQP). These ratios are used to express the differential activity of specific pathway enzymatic steps/branches. KYN/TRP is a proxy of the activity of the first step of the pathway, QUIN/KYNA is often called the “neurotoxic ratio” and can be viewed as a proxy for the glutamate agonism of QUIN *vs* the antagonist KYNA, and QUIN/PIC is a proxy for ACMSD activity, the enzyme that produces PIC at the expense of QUIN (Fig. 1).

### Statistical analysis

Statistical analyses were conducted using RStudio 4.0.2 (https://cran.r-project.org/). The sample size was calculated to ensure adequate power to detect a prespecified effect size of ≥ 0.8 based on kynurenine metabolite measurements in plasma from psychiatric patients [23]. Concentrations of biomarkers were natural log-transformed when applicable to meet model assumptions. The following number of individuals without missing data for at least one of the analyses were included at each timepoint: 112 (1st trimester), 88 (2nd trimester), 82 (3rd trimester), 78 (postpartum). 56 women had data from all timepoints.

Associations between depressive symptoms and biomarkers across time were evaluated by ordinal mixed-effects regression, and logistic mixed-effects regression was used to evaluate the risk of experiencing significant depressive symptoms (binary outcome: EPDS ≥ 13). Bayesian generalized (non)-linear ordinal multilevel models and Bayesian logistic regression [24], both adjusted for age, were used to predict subsequent EPDS scores (ordinal) and EPDS ≥ 13 (binary) respectively. For both ordinal and logistic analyses, a full model comprised of the principal components that explained ≥ 10% of the markers’ variance and a horseshoe prior was initially fit to maximize predictive capacity while avoiding multicollinearity and overfitting. Model performance was evaluated via leave-one-out (LOO) cross-validation by comparing the full model with age-only and null, intercept-only models [25]. Bayesian ordinal and logistic models with flat priors were also built for each of the top markers in the first principal component to compare with the full model. Predictive accuracy was visualized by ROC and PR curves [26]. Marginal effect plots from individual biomarkers by age were generated based on the Bayesian models to visualize predicted PPD diagnoses.

## Results

### Characteristics of the cohort

The demographic characteristics of the participants are summarized in Table 1. The average age was 26 years (18–44 range). 37 out of the 114 women (32%) experienced significant depressive symptoms (EPDS ≥ 13) at one or more timepoints and 12 women (10.5%) received a formal diagnosis of PPD via DSM SCID interview during the study. The percentage of women experiencing significant depressive symptoms (EPDS score of ≥ 13) was 19.3%, 13.6%, 15.8%, and 14.1% during the 1st, 2nd, and 3rd trimester, and in the postpartum, respectively. Depressive symptoms and the median levels of the biomarkers at each timepoint are shown in Table. S1.

### Biomarkers significantly correlated with depression severity and risk

IL1*β*, IL-6 and QUIN, were significantly associated with depression severity and/or higher odds of having an EPDS ≥ 13. Higher levels of IL-6 were found to be associated with increased depression severity and risk of experiencing significant depressive symptoms at all timepoints (*P* = 0.01 Table. S2). Increasing levels of IL-1*β* were further associated with worsening depressive symptoms (EPDS, ordinal regression *P* = 0.02) and an elevated risk of experiencing significant depressive symptoms (EPDS ≥ 13, logistic regression *P* = 0.01) with an OR increase of 32.3% (95% CI: 7.0, 63.6) per median absolute deviation (MAD) of IL-1*β* (Table. S2). The association between QUIN and depression was also robust, as higher levels in the 3rd trimester were associated with both increasing depression severity (*P* = 0.04, EPDS score) and risk of falling into the category of significant symptoms (EPDS ≥ 13) with an OR increase 41.5% per MAD; 95% CI: 1.8, 96.6; *P* = 0.02 (Table. S2).

### Principle component analysis (PCA)

We assessed the capacity of the biomarkers at one timepoint to predict depressive symptoms at the following timepoint Table 2. To prevent multicollinearity among all the biomarkers from influencing the model, a PCA was conducted to reduce the number of predictors into a smaller set of independent covariates. All principal components (PCs) that explained >10% of the total variance among all markers (6 cytokines, 4 kynurenine metabolites, tryptophan, serotonin and 3 derivative ratios) were included in the final model (PC1 to PC5). The top contributors to PC1 were TNF, rKT, QUIN, KYN, rQK, IL-10, IL-6, rQP; they cumulatively explain >90% of the variance of PC1 (Table 2). These markers were included in the subsequent analyses.

**Table 2:** Biomarker contribution to -and correlation with- PC1 during the 2nd trimester.

Biomarker	Contribution to PC1 (%)	Weight to PC1 (MAD)	PC1 Spearman Rho (FDR adjusted *p* value)	3rd trimester acute EPDS: Percent change in OR (95% credible interval)			
				EPDS	Posterior probability	EPDS ≥ 13	Posterior probability
TNF	18.67	0.54	0.789 (<0.0001)	**53.7 (18.5, 105.4)**	**>0.99**	**33.6 (0, 94.6)**	**0.95**
rKT^a^	18.12	0.53	0.768 (<0.0001)	**51.7 (22.1, 87.8)**	**>0.99**	**52.2 (12.7, 116.0)**	**0.99**
QUIN	15.17	0.48	0.713 (<0.0001)	**78.6 (28.4, 150.9)**	**>0.99**	**91.6 (15.0, 232.0)**	**0.98**
KYN	14.23	0.47	0.572 (<0.0001)	**56.8 (23.4, 99.4)**	**>0.99**	**52.2 (8.3, 120.3)**	**0.98**
rQK	8.63	0.36	0.652 (<0.0001)	**68.2 (138.8, 146.0)**	**0.99**	**124.8 (17.4, 357.2)**	**0.98**
IL-10	5.9	0.3	0.330 (0.0022)	5.1 (−5.8, 18.5)	0.76	5.1 (−12.2, 25.9)	0.72
IL-6	5.52	0.29	0.465 (<0.0001)	**64.9 (32.3, 107.5)**	**>0.99**	**58.4 (22.1, 111.7)**	**>0.99**
rQP	5.3	0.29	0.639 (<0.0001)	19.7 (−16.5, 73.3)	0.79	19.7 (−22.1, 80.4)	0.78

Correlation between biomarkers in the 2nd trimester and EPDS scores from the 3rd trimester via Bayesian’s prediction models. Biomarkers were standardized via Robust Standardization. One PC1 unit corresponds to a 0.54 MAD (median absolute deviation) change in TNF, a 0.53 MAD change in rKT, a 0.48 MAD change in QUIN, a 0.47 MAD change in KYN, a 0.36 MAD change in rQK, a 0.3 MAD change in IL-10, a 0.29 MAD change in IL-6, and a 0.29 MAD change in rQP.

^a^rKT: odds ratio from 1/100 increase in rKT.

Bolded text indicates >95% chance marker has a true association with EPDS or EPDS>=13.

### Prediction of depressive symptoms utilizing individual markers

IL-6, rKT, QUIN, and KYN measured in the 2nd trimester have strong evidence (>95% chance) of being positively associated with both depression severity (EPDS) and risk of significant depressive symptoms (EPDS ≥ 13) in the 3rd trimester. Also, TNF and rQK in the 2nd trimester, were strongly associated with depression severity in the 3rd trimester (EPDS) (Table 2).

In the ROC analysis, IL-6 had the best performance among the individual biomarkers used to predict depressive symptoms (AUC = 0.79 and 0.8 by Bayesian ordinal and logistic regression, respectively). Kynurenine, QUIN and rKT also produced good predictions with six out of seven markers having a ROC AUC > 0.7 by either model (Fig. S1A, C). Precision recall analyses were also estimated and confirmed the predictive value of our model (Fig. S1B, D).

### Prediction of depression severity at future timepoints using multiple markers

To estimate the performance of our predictive algorithm on data not used to train our model, we first performed a leave-one-out cross validation, which indicated that our predictability, as could be generalized to other cohorts from this data set, would be optimal from mid- to late pregnancy (2nd to 3rd trimester, see Table. S3).

**Fig. 2:**
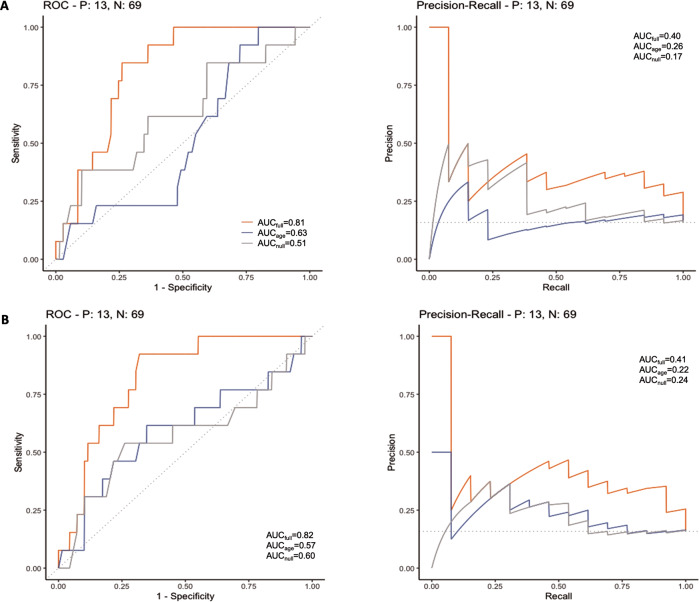
ROC and PR curves of the prediction model comparisons. ROC and PR curves of the prediction model comparisons (between full, age- only and null models) using biomarkers collected during the 2nd trimester to predict 3rd trimester EPDS ≥ 13. **A** using ordinal regression (cumulative probability that EPDS ≥ 13), **B** logistic regression (estimated probability EPDS ≥ 13). The full model outperforms the other two models in both the ROC and PR curves. The performance of the PR curve is important when the number of cases (EPDS ≥ 13) is not equal to non-cases (EPDS < 13). The leave-one-out cross-validation results are also consistent with the full model having the best predictive accuracy.

The prediction from 2nd to 3rd trimester is illustrated in Fig. 2 (ROC- and PR curves). For both models (predicting EPDS and EPDS ≥ 13), the first principal component (PC1) had >99% chance of being positively associated with depression severity and >94% for the depressive phenotype (Bayes Factors = 265 and 17, respectively). For each unit increase in PC1, the odds of experiencing significant depressive symptoms increased by 31% (95% CI: ~0, 77; Table 2). Based on the area under ROC- and PR curves, the full model nominally outperformed each individual marker for predicting risk of significant depressive symptoms. Both ordinal and logistic regression full models had ROC AUC = 0.83, PR AUC = 0.41 (Fig. 2).

### PPD diagnosis and usefulness of biological “depression-warning” triggers

**Fig. 3:**
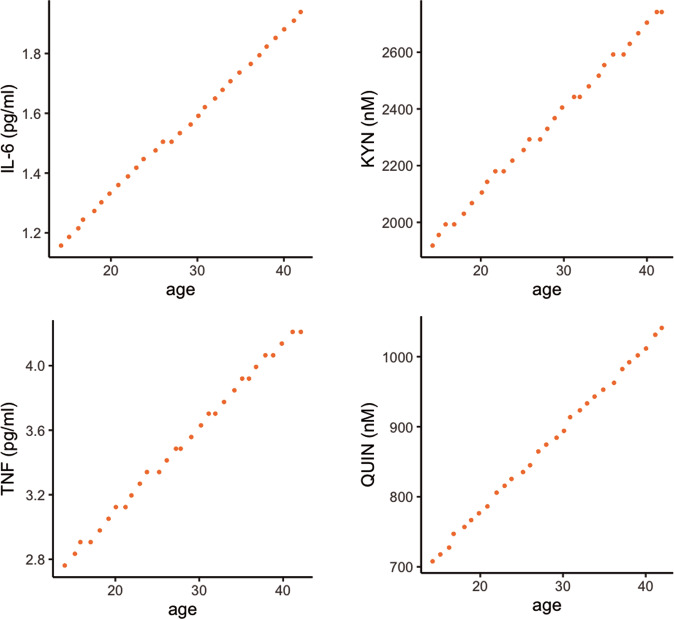
Depression-warning cut off per age. Bayesian models with flat priors were used to predict the probability of each 3rd trimester EPDS score based on individual marker levels and age. Plotted here are the minimal marker levels where the most probable EPDS score is ≥10 for women ages 18–44. As marker levels exceed these plotted thresholds, the probability that a given woman would score ≥ 10 on the EPDS scale in the 3^rd^ trimester continues to increase asymptotically to 1. An EPDS score of 10 or greater is used clinically to screen women for PPD, thus these plots present a proof-of-concept for an advanced screening method where a woman’s age and measures of these 4 markers could jointly be used to flag someone as likely to score ≥ 10 on the EPDS assessment in the next trimester and therefore considered to be at risk of developing future PPD.

As a proof-of-concept pilot, we established whether 12 patients diagnosed with PPD using SCID interviews during pregnancy would have been identified using our proposed biomarker panel, derived from the 2nd trimester, where we found most evidence for generalizability. To this end, we generated an age-adjusted “depression-warning” level for the four main risk biomarkers identified in our statistical models (IL-6, TNF, kynurenine and QUIN). The biomarker level chosen to trigger “depression-warning” was set as the level where the probable EPDS score was >10 at each age of the women, the same level that triggered a SCID diagnostic interview in this study. The depression-warning levels per age for IL-6, TNF, QUIN and kynurenine are visualized in Fig. 3. Using these biological “depression-warning” measurements, we would have identified 83.3% of all SCID diagnosed cases, based on only the biomarker measures. Adding the rKT ratio to the biomarkers did not further increase the test’s sensitivity.

## Discussion

We established that inflammatory cytokines, in particular IL-6 and IL-1*β*, as well as kynurenine pathway metabolites, are associated with the severity of depressive symptoms in pregnancy and the postpartum, and that these markers were useful to predict the future development of depression during pregnancy. Our full model, composed of all 15 biomarkers (including three ratios), had an AUC of more than 83% for predicting depression as defined by EPDS ≥13. Moreover, IL-6, QUIN, TNF, and KYN showed strong individual accuracy in predicting depression risk and severity at future timepoints, with the best performance using the 2nd trimester markers to predict symptoms emerging in the 3rd trimester. In our proof-of-concept pilot, we found that we would have identified 83% of PDD-diagnosed women (identified by SCID interviews) by means of the biomarkers only. Certainly, further evaluation in larger cohorts will be necessary to refine and fully estimate the specificity, sensitivity, and positive predictive value of these biomarkers. Additionally, the analytical methods and reagents need to be cross-validated and standardized across multiple sites to allow for any broad implementation.

During pregnancy and postpartum women experience major immune function fluctuations. These immunological changes are part of the women’s adaptation to the fetal development and are critical for both maternal and infant health. During a normal pregnancy, the maternal immune system goes through several physiological stages, including an early, more pro-inflammatory state, important for implantation and placentation, characterized by increased production of cytokines, chemokines and growth factors; next, comes an anti-inflammatory phase, characterized by rapid fetal growth; and lastly, another pro-inflammatory phase, preceding delivery in preparation for parturition [27]. During the postpartum phase, the body gradually returns to a nonpregnant homeostasis, as it relates to immune regulation [28]. The physiological fluctuations in inflammatory factors can impact emotion and behavior, and may do so in pregnancy [29]. However, we propose that when these changes become extreme, or take place in women vulnerable to inflammation-induced depression, the mood changes can contribute to the pathophysiological processes leading to PPD. It is interesting to note that the markers we examined here showed predictive capacity during pregnancy, but not for the postpartum. This likely indicates that partially differential mechanisms, such as hormonal changes linked to parturition and possibly lactation, also influence the development of depression during this period [30].

In addition to their role as biomarkers of disease, the analytes measured in this study have important biological functions that can be part of the molecular underpinnings of depressive symptoms. The metabolites of the kynurenine pathway have both immunoregulatory, inflammatory and neuroactive properties [18]. QUIN, in particular, was a significant predictor of future depressive symptoms from mid- to late pregnancy. This metabolite is an endogenous modulator of glutamate receptors, with agonist properties on the *N*-methyl-D-aspartate receptor (NMDAR) [17]. QUIN also has additional effects, including increasing glutamate release while inhibiting its reuptake by astrocytes, both of which have stimulatory effects on glutamatergic neurotransmission [31]. Peripheral levels of cytokines and kynurenine metabolites are known to reflect the central levels, as our group and others have shown significant correlations between the blood and CSF levels of acute-phase reactants, inflammatory cytokines and kynurenine metabolites, including QUIN [32, 33]. Although in this study, we did not draw any CSF samples from the pregnant women, several studies have shown evidence that peripheral inflammation and kynurenine pathway activation are closely mirrored in the central compartment [32, 34, 35]. Therefore, it is plausible that the increased inflammation and pathway activation we observe in the periphery, could reflect central mechanisms underlying symptom generation.

Previous studies in depression and suicidality have found increased levels of cytokines, in particular IL-6, and kynurenine metabolites, in blood and CSF of patients with depression and suicidality [36]. Additionally, experimental studies confirmed that alterations in inflammation and kynurenine metabolism can lead to depressive-like behavior. This has been established in animal models [37] and human volunteers injected with endotoxin to stimulate inflammation [38]. It is known that interferon-*α* injections, to treat hepatitis C, lead to depressive symptoms, together with an increase in cytokines and QUIN in the CSF [35]. Interestingly, in an experimental study using mice, depressive-like effects of inflammation were fully reversed by modulating the NMDAR following ketamine treatment [39]. In the current study, the finding that these biomarkers not only correlate with depressive symptoms, but also might predict future symptom development, further strengthens the causal inference that these metabolites and cytokines might contribute to the development of depressive symptoms, in addition to being biomarkers of disease. Consequently, these mechanisms could be considered in the future therapeutic development for PPD. As such, it will also be important to understand the factors contributing to the inflammation and kynurenine pathway activation that we observe in women with PPD. Examples of contributing mechanisms could include lack of vitamin D, infections, changes in gonadal hormones or cortisol [40], as well as dietary factors [41]. Modulating the upstream causes of inflammation may be feasible in certain women, while treating the downstream pathways triggered by inflammation might be optimal in others.

To our knowledge, this is the first longitudinal study examining the ability of a panel of mechanistically implicated cytokines and tryptophan metabolites to predict the development of depression in pregnancy. Predicting which individuals may be at risk for depression during pregnancy is a difficult task for clinicians. The importance of detecting depression in pregnant women has been highlighted by American College of Obstetricians and Gynecologists [42], which encourages self-assessments using the EPDS when women are in contact with health care professionals during and after pregnancy. Reporting of depressive symptoms in this period may also be linked with feelings of shame or guilt due to cultural stigma, leading to an underestimation of symptom prevalence and severity and reduced help-seeking [43]. Importantly, the addition of biological markers as tools to predict future depression risk, acting as “depression-warning” signs, could help clinicians schedule more frequent follow-up visits, consider stress-reduction and psychotherapy, include supportive or cognitive-behavioral techniques, or possibly intervene with preventative measures (e.g., use of anti-inflammatories) before worrisome symptoms begin.

Limitations of this study include that it was conducted at a single center, which could reduce generalizability. It is however important to note that our cohort was relatively diverse, <50% White/Caucasian (Table 1). Also, the sample size was moderate for a study of this type (>350 samples were analyzed, over four timepoints). Therefore, these findings warrant further replication in larger cohorts to establish whether the biomarkers can be developed into a clinically useful predictive panel for depression risk in pregnant women. Additional biomarkers, possibly including neopterin, cortisol and additional cytokines, could also be assayed to determine whether the accuracy of prediction could be improved even further. Despite the limitations, our current study provides important insights into the possible role of inflammatory biomarkers in perinatal depression.

In conclusion, we established that inflammatory cytokines and kynurenine metabolites are associated with depression severity during pregnancy and the postpartum. Together with the data from both clinical and animal models demonstrating a causal role for these biological mediators, inducing depression and depressive-like behavior [44, 45], our longitudinal data suggest that our analytes could also be causal mediators in pregnancy-related depression. Randomized controlled experiments are necessary to confirm this hypothesis, as an initial step in assessing the possibility of treating inflammation in PPD; caution must however be taken due to the involvement of these factors in fetal development. Importantly, our study showed that a set of biomarkers was able to predict the future development of depression during pregnancy, and steps could be taken to move these markers into clinical risk assessments.

## Supplementary information

Raw data of EPDS and biomarker levels across all visits

Table of biomarkers are associated with depression severity

Leave-one-out cross-validation

ROC and PR curves for the individual makers
