# Supplementary material for: Cytokines and tryptophan metabolites can predict depressive symptoms in pregnancy
Source: Transl Psychiatry. 2022 Jan 26;12:35. doi: 10.1038/s41398-022-01801-8 (PMC8789799; doi:10.1038/s41398-022-01801-8)
Supplement: Supplementary file 1 — Raw data of EPDS and biomarker levels across all visits [file 41398_2022_1801_MOESM1_ESM.docx]

**Table S1. Raw data of EPDS and biomarker levels across all visits.**

|  | **1^st^ trimester** | | **2^nd^ trimester** | | **3^rd^ trimester** | | **Post-partum** | |
| --- | --- | --- | --- | --- | --- | --- | --- | --- |
|  | $\boldsymbol{<}$**13** | $\boldsymbol{\geq}$**13** | $\boldsymbol{<}$**13** | $\boldsymbol{\geq}$**13** | $\boldsymbol{<}$**13** | $\boldsymbol{\geq}$**13** | $\boldsymbol{<}$**13** | $\boldsymbol{\geq}$**13** |
| EDPS | 4.5  2-8 | 14.5  13-19 | 4.5  2-8 | 16  15-18 | 4  1-8 | 15  13-17 | 4  2-6 | 14.5  13-17 |
| IL-1𝛽 (pg/ml) | 0.06  0.04-0.08 | 0.09  0.05-0.11 | 0.05  0.04-0.07 | 0.10  0.06-0.14 | 0.07  0.04-0.09 | 0.09  0.05-0.11 | 0.08  0.06-0.12 | 0.13  0.08-0.24 |
| IL-2 (pg/ml) | 0.16  0.12-0.28 | 0.34  0.12-0.45 | 0.15  0.12-0.38 | 0.15  0.12-0.45 | 0.18  0.12-0.29 | 0.27  0.12-0.60 | 0.23  0.12-0.57 | 0.22  0.13-0.33 |
| IL-6 (pg/ml) | 0.64  0.46-0.92 | 0.60  0.44-1.41 | 0.60  0.40-1.11 | 0.71  0.55-1.38 | 0.75  0.56-1.10 | 1.19  0.84-1.89 | 0.73  0.51-1.22 | 0.89  0.64-1.17 |
| IL-8 (pg/ml) | 2.8  2.0-4.2 | 2.9  2.2-3.6 | 2.4  1.9-3.0 | 2.9  2.5-4.4 | 2.5  2.2-3.2 | 3.1  2.5-4.5 | 4.2  3.4-5.3 | 5.1  3.4-6.7 |
| IL-10 (pg/ml) | 0.33  0.23-0.41 | 0.36  0.27-0.41 | 0.34  0.27-0.45 | 0.40  0.30-0.51 | 0.34  0.27-0.44 | 0.37  0.28-0.55 | 0.30  0.25-0.39 | 0.31  0.24-0.37 |
| TNF (pg/ml) | 1.84  1.60-2.32 | 1.84  1.70-2.22 | 1.94  1.59-2.25 | 2.29  1.75-2.63 | 2.0  1.82-2.44 | 2.6  1.79-3.5 | 2.5  2.0-2.9 | 2.5  2.1-3.4 |
| TRP (µM) | 32.85  30.46-37.69 | 34.75  30.49-37.27 | 31.52  28.12-34.6 | 33.29  28.11-40.75 | 28.35  25.33-31.13 | 30.91  23.93-34.83 | 36.02  32.47-42.67 | 39.28  34.34-41.93 |
| SERO (nM) | 49.5  28.0-83.9 | 41.2  19.2-70.3 | 35.8  20.3-68.1 | 49.2  27.9-172 | 28.4  16.3-70.1 | 23.4  9.9-52.8 | 77.3  42.6-175.2 | 66.0  42.8-103.2 |
| QUIN (µM) | 0.43  0.35-0.54 | 0.41  0.35-0.46 | 0.53  0.42-0.61 | 0.66  0.45-0.80 | 0.61  0.51-0.74 | 0.71  0.60-1.26 | 0.65  0.51-0.79 | 0.69  0.53-0.75 |
| PIC  (µM) | 0.64  0.53-0.86 | 0.72  0.45-0.95 | 0.69  0.56-0.93 | 0.70  0.55-1.08 | 0.73  0.59-1.02 | 0.83  0.64-1.07 | 0.57  0.45-0.68 | 0.62  0.47-0.69 |
| KYNA (nM) | 44.8  32.8-55.8 | 41.4  34.0-49.3 | 41.5  32.6-54.0 | 44.6  28.0-63.8 | 40.6  27.1-52.9 | 40.2  34.2-59.8 | 60.5  44.1-81.8 | 52.1  42.5-68.4 |
| KYN (µM) | 1.34  1.15-1.59 | 1.43  1.20-1.56 | 1.38  1.17-1.62 | 1.62  1.44-1.96 | 1.32  1.22-1.46 | 1.68  1.30-1.90 | 2.11  1.81-2.39 | 1.99  1.64-2.46 |

Raw values, Median + IQR. The groups included in the table comprise data from subjects that scored below 13 at each timepoint on the EPDS ($<$13**)** and those who scored 13 and above ($\boldsymbol{\geq}$13).
