# Supplementary material for: Cytokines and tryptophan metabolites can predict depressive symptoms in pregnancy
Source: Transl Psychiatry. 2022 Jan 26;12:35. doi: 10.1038/s41398-022-01801-8 (PMC8789799; doi:10.1038/s41398-022-01801-8)
Supplement: Supplementary file 2 — Table of biomarkers are associated with depression severity [file 41398_2022_1801_MOESM2_ESM.docx]

**Table S2.** **Table of biomarkers are associated with depression severity**

|  | | Total EPDS | | |  | EPDS$\geq$13 | | |
| --- | --- | --- | --- | --- | --- | --- | --- | --- |
| Biomarker | Time point | Odds ratio  (%) | 95% CI (%) | P |  | Odds ratio  (%) | 95% CI (%) | P |
| **IL-1β** | All | **17.9** | **2.6, 35.5** | **0.021^*^** |  | **32.3** | **7.0, 63.6** | **0.01^**^** |
| IL2 | All | -0.3 | -2.8, 2.4 | 0.843 |  | -4.5 | -13.9, 5.9 | 0.308 |
| **IL-6** | **All** | **20.1** | **4.1, 38.4** | **0.012^*^** |  | **31.7** | **6.1, 63.6** | **0.013^*^** |
| IL-8 | All | 8.3 |  | 0.098 |  | -23.9 |  | 0.266 |
|  | 1^st^ | 8.3 | -1.5, 19 | 0.449 |  | -23.9 | -53, 23.2 | 0.267 |
|  | 2^nd^ | 18.2 | -2.4, 43.2 | 0.088 |  | 57.0 | -1.7, 150.7 | 0.059 |
|  | 3^rd^ | -1.0 | -29.8, 39.7 | 0.955 |  | 55.4 | -10.2, 169.4 | 0.115 |
|  | PP | -8.5 | -32.4, 23.9 | 0.563 |  | 21.3 | -28.1, 104.4 | 0.470 |
| IL-10^*^ | All | 3.9 |  | 0.549 |  | -2.8 |  | 0.819 |
|  | 1^st^ | 3.9 | -8.4, 17.9 | 0.549 |  | -2.8 | -23.8, 24.0 | 0.819 |
|  | 2^nd^ | -8.9 | -19.2, 2.8 | 0.129 |  | -5.4 | -33.4, 34.4 | 0.758 |
|  | 3^rd^ | 15.3 | -0.9, 34.1 | 0.066 |  | 6.8 | -10.8, 27.8 | 0.472 |
|  | PP | 1.1 | -3.2, 5.6 | 0.622 |  | -2.1 | -17.1, 15.6 | 0.804 |
| TNF | All | 11.9 | -10.7, 40.1 | 0.328 |  | 19.7 | -11.8, 62.6 | 0.249 |
| TRY | All | 2.5 | -20.9, 32.8 | 0.853 |  | 29.4 | -13.5, 93.7 | 0.210 |
| SERO | All | -1.5 | -8.7, 6.2 | 0.69 |  | 0.1 | -11.8, 13.8 | 0.983 |
| KYN^*^ | All | -22.7 |  | 0.276 |  | -4.8 |  | 0.891 |
|  | 1^st^ | -22.7 | -51.3, 22.9 | 0.276 |  | -4.8 | -53.1, 92.9 | 0.891 |
|  | 2^nd^ | 29.7 | -11.4, 89.8 | 0.180 |  | 76.3 | -8.7, 240.4 | 0.092 |
|  | 3^rd^ | 78.6 | -8.0, 246.2 | 0.086 |  | **256.6** | **21.3, 948.6** | **0.021^*^** |
|  | PP | 34.3 | -3.9, 87.8 | 0.085 |  | -5.0 | -46.8, 69.9 | 0.863 |
| KYNA | All | -0.5 | -23.1, 28.8 | 0.969 |  | -3.8 | -35.0, 42.3 | 0.846 |
| **QUIN^*^** | All | -12.3 |  | 0.563 |  | -18.6 |  | 0.560 |
|  | 1^st^ | -12.3 | -43.7, 36.6 | 0.662 |  | -18.6 | -59.3, 62.7 | 0.561 |
|  | 2^nd^ | 20.2 | -22.6, 86.8 | 0.413 |  | 47.4 | -28.2, 202.2 | 0.290 |
|  | **3^rd^** | **41.5** | **1.8, 96.6** | **0.039^*^** |  | **98.2** | **10.4, 255.7** | **0.022^*^** |
|  | PP | 22.5 | -18.1, 83.3 | 0.324 |  | -15.4 | -60.7, 82.0 | 0.669 |
| PICO | All | 0.6 | -16.4, 20.9 | 0.954 |  | -4.5 | -30.6, 31.3 | 0.775 |
| rKT^1^ | All | 9.2 | -9.7, 32.0 | 0.362 |  | 10.1 | -16.1, 44.5 | 0.487 |
| rQK | All | 40.7 |  | 0.143 |  | 1.4 |  | 0.967 |
|  | 1^st^ | 40.7 | -10.9, 122.1 | 0.143 |  | 1.4 | -47.4, 95.4 | 0.967 |
|  | 2^nd^ | 21.9 | -17.7, 80.6 | 0.322 |  | 15.1 | -40.4, 122.6 | 0.676 |
|  | 3^rd^ | 32.0 | -7.5, 88.7 | 0.127 |  | 57.1 | -11.8, 180.1 | 0.124 |
|  | PP | -14.5 | -42.8, 27.8 | 0.444 |  | 13.8 | -44.1, 131.6 | 0.722 |
| rQP | All | 14.0 | -6.4, 39.0 | 0.192 |  | 16.8 | -13.3, 57.3 | 0.308 |

Total EPDS, ordinal mixed-effects regression and/or risk of being diagnosed with the depressive phenotype (EPDS ≥ 13, logistic mixed-effects regression) across each timepoint, adjusted for age. Markers were normalized via robust standardization prior to analysis. ^*^Models include the interaction between biomarker and time point.
