# Supplementary material for: Cytokines and tryptophan metabolites can predict depressive symptoms in pregnancy
Source: Transl Psychiatry. 2022 Jan 26;12:35. doi: 10.1038/s41398-022-01801-8 (PMC8789799; doi:10.1038/s41398-022-01801-8)
Supplement: Supplementary file 3 — Leave-one-out cross-validation [file 41398_2022_1801_MOESM3_ESM.docx]

**Table S3. Leave-one-out cross-validation**

| Prediction period | Model | Elpd^1^_diff | Se^2^_diff |
| --- | --- | --- | --- |
| 1^st^ -> 2^nd^ EPDS | Null | 0.0 | 0.0 |
|  | Age only | -0.3 | 0.4 |
|  | Full | -0.8 | 0.5 |
| 1^st^ -> 3^rd^ EPDS | Age only | 0.0 | 0.0 |
|  | Null | -0.3 | 1.2 |
|  | Full | -1.2 | 0.6 |
| 1^st^ -> post-partum EPDS | Age only | 0.0 | 0.0 |
|  | Null | -1.2 | 2.1 |
|  | Full | -1.4 | 0.8 |
| **2^nd^ -> 3^rd^ EPDS** | **Full** | **0.0** | **0.0** |
|  | Age only | -3.9 | 3.0 |
|  | Null | -4.1 | 2.7 |
| 2^nd^ -> post-partum EPDS | Age only | 0.0 | 0.0 |
|  | Full | -1.9 | 1.0 |
|  | Null | -2.4 | 2.2 |
| 3^rd^ -> post-partum EPDS | Age only | 0.0 | 0.0 |
|  | Full | -1.7 | 1.0 |
|  | Null | -1.8 | 2.2 |

Leave one out cross validation of the full models by ordinal regression at different time points comparing predictive accuracy to age-only and null (mean only) models. ELPD: expected log pointwise predictive density SE: standard error of ELPD.
