# Supplementary figures and images for: Cytokines and tryptophan metabolites can predict depressive symptoms in pregnancy
Source: Transl Psychiatry. 2022 Jan 26;12:35. doi: 10.1038/s41398-022-01801-8 (PMC8789799; doi:10.1038/s41398-022-01801-8)

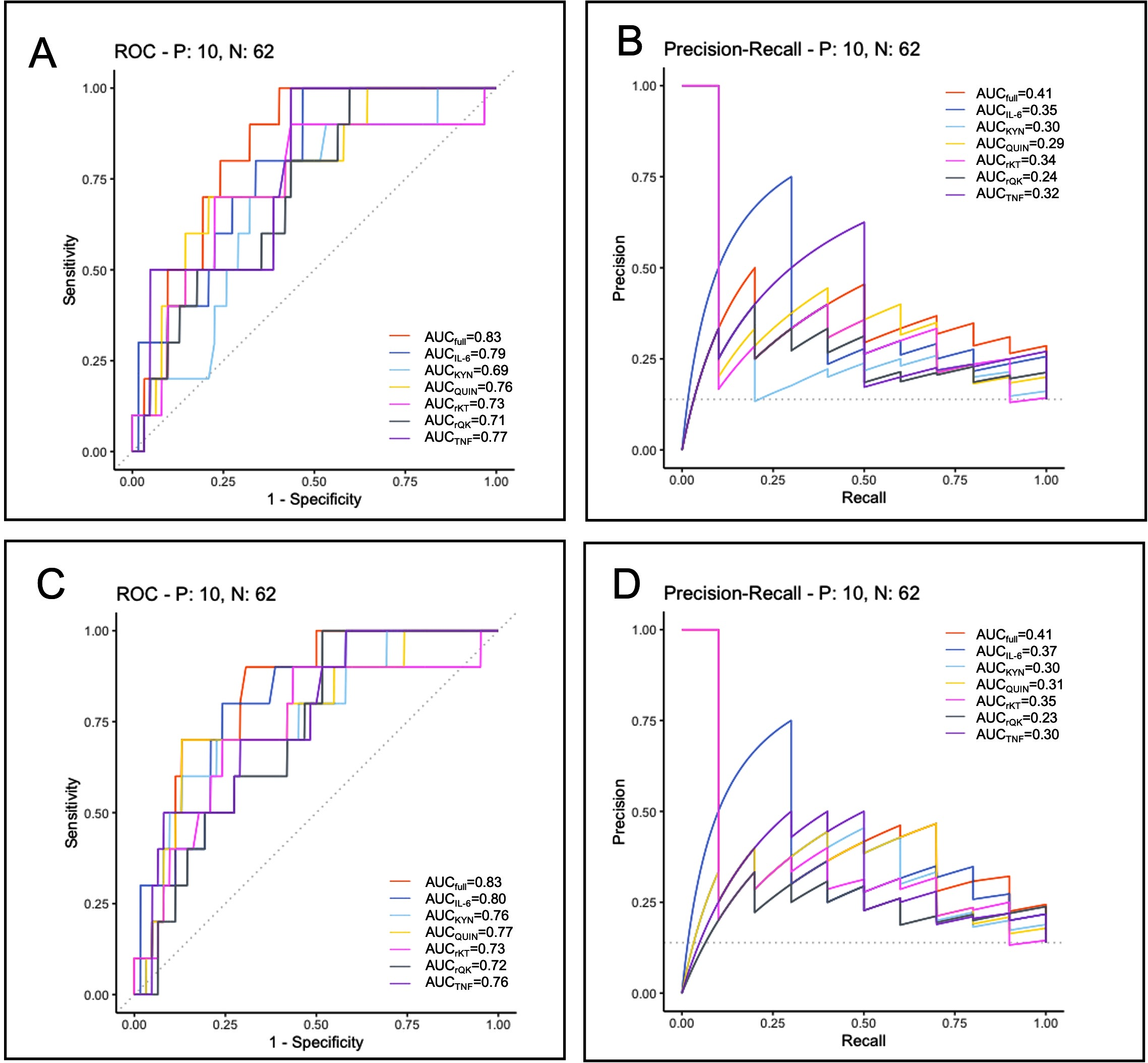

Supplement: Supplementary file 4 — ROC and PR curves for the individual makers [file 41398_2022_1801_MOESM4_ESM.tif]
